# Effects of an elemental diet to reduce adverse events in patients with esophageal cancer receiving docetaxel/cisplatin/5-fluorouracil: a phase III randomized controlled trial—EPOC 2 (JFMC49-1601-C5)☆

**DOI:** 10.1016/j.esmoop.2021.100277

**Published:** 2021-10-06

**Authors:** Y. Tanaka, H. Takeuchi, Y. Nakashima, H. Nagano, T. Ueno, K. Tomizuka, S. Morita, Y. Emi, Y. Hamai, J. Hihara, H. Saeki, E. Oki, C. Kunisaki, E. Otsuji, H. Baba, H. Matsubara, Y. Maehara, Y. Kitagawa, K. Yoshida

**Affiliations:** 1 Department of Surgical Oncology, Graduate School of Medicine, Gifu University, Gifu, Japan; 2 Department of Surgery, School of Medicine, Hamamatsu University, Hamamatsu, Japan; 3 Department of Surgery and Science, Graduate School of Medical Sciences, Kyushu University, Fukuoka, Japan; 4 Gastroenterological, Breast and Endocrine Surgery, Graduate School of Medicine, Yamaguchi University, Yamaguchi, Japan; 5 Department of Dentistry, National Cancer Center Hospital, Tokyo, Japan; 6 Department of Dentistry, Cancer Institute Hospital of JFCR, Tokyo, Japan; 7 Department of Biomedical Statistics and Bioinformatics, Graduate School of Medicine, Kyoto University, Kyoto, Japan; 8 Department of Surgery, Saiseikai Fukuoka General Hospital, Fukuoka, Japan; 9 Department of Surgical Oncology, Hiroshima University Hospital, Hiroshima, Japan; 10 Department of Surgery, Hiroshima City Asa Citizens Hospital, Hiroshima, Japan; 11 Department of General Surgical Science, Gunma University, Maebashi, Japan; 12 Department of Surgery, Gastroenterological Center, Yokohama City University, Yokohama, Japan; 13 Department of Surgery, Kyoto Prefectural University of Medicine, Kyoto, Japan; 14 Department of Gastroenterological Surgery, Kumamoto University, Kumamoto, Japan; 15 Department of Frontier Surgery, Graduate School of Medicine, Chiba University, Chiba, Japan; 16 Director, Kyushu Central Hospital of the Mutual Aid Association of Public School Teachers, Fukuoka, Japan; 17 Department of Surgery, Keio University School of Medicine, Tokyo, Japan

**Keywords:** oral mucositis, elemental diet, chemotherapy, central review system, esophageal cancer

## Abstract

**Background:**

Oral mucositis (OM) is an unpleasant adverse event in patients receiving chemotherapy. A prospective feasibility study showed that elemental diet (ED), an oral supplement that does not require digestion, may prevent OM. Based on this, we established a central review system for oral cavity assessment by dental oncology specialists blinded to background data. We used this system to elucidate the preventive effect of an ED against OM in patients with esophageal cancer receiving docetaxel, cisplatin, and 5-fluorouracil (DCF) therapy.

**Patients and methods:**

In this phase III, multicenter, parallel-group, controlled trial, patients consuming a normal diet orally were randomly assigned (1 : 1) to receive two cycles of DCF with (group A) or without (group B) an ED (Elental® 160 g/day). We assessed the incidence of grade ≥2 OM evaluated by two reviewers, changes in body weight, prealbumin, C-reactive protein, and DCF completion rate based on ED compliance.

**Results:**

Of the 117 patients randomly assigned to treatment, four failed to start treatment and were excluded from the primary analysis; thus, groups A and B comprised 55 and 58 patients, respectively. There were no significant differences in background characteristics. Grade ≥2 OM was observed in eight (15%) and 20 (34%) patients in groups A and B, respectively (*P* = 0.0141). Changes in body weight and prealbumin during the two DCF cycles were significantly higher in group A than B (*P* = 0.0022 and 0.0203, respectively). During the first cycle, changes in C-reactive protein were significantly lower in group A than B (*P* = 0.0338). In group A (receiving ED), the DCF completion rate was 100% in patients with 100% ED compliance and 70% in patients failing ED completion (*P* = 0.0046).

**Conclusions:**

The study findings demonstrate that an ED can prevent OM in patients with esophageal cancer receiving chemotherapy.

## Highlights

• Patients receiving docetaxel, cisplatin, and 5-fluorouracil (DCF) chemotherapy commonly develop oral mucositis (OM).
• An elemental diet (ED) was able to prevent OM in patients with esophageal cancer receiving DCF.
• Grade ≥2 OM was observed in 15% of patients receiving the ED versus 34% of those not receiving the ED (*P* = 0.0141).
• Body weight was maintained in the ED group, and hematologic toxicities were lower, compared with the non-ED group.
• The DCF completion rate significantly correlated with ED compliance (*P* = 0.0046).

## Introduction

Oral mucositis (OM) is a commonly occurring adverse event (AE) in cancer patients. OM may lead to oral pain, refusal to eat, weight loss, infection, and systemic spread of local inflammation.^1^ Severe OM in cancer patients can delay cancer treatment and worsen prognosis.^1^ Suppressing the development of OM can enhance chemotherapy continuity.^2^

Studies showed that OM develops in 5%-50% of patients receiving standard-dose chemotherapy and 42%-98% receiving high-dose chemotherapy such as hematopoietic stem cell transplantation.^3^, ^4^, ^5^, ^6^, ^7^, ^8^, ^9^ The incidence varies by cancer type and chemotherapeutic regimen,^10^, ^11^, ^12^, ^13^ with particularly high OM frequencies reported for docetaxel, cisplatin, and 5-fluorouracil (DCF; 86%); 5-fluorouracil, leucovorin, and irinotecan (80%); and 5-fluorouracil, cyclophosphamide, and doxorubicin (79%).^14^

It has been reported that oral glutamine administration can reduce OM in patients receiving chemotherapy,^15^ although meta-analyses have not supported this finding.^16^ It is possible that due to intestinal absorption issues, glutamine administration alone may be insufficient to prevent the development of OM.^17^^,^^18^ The oral elemental diet (ED) (Elental®; EA Pharma Co., Ltd, Tokyo, Japan; Supplementary Material S1, available at https://doi.org/10.1016/j.esmoop.2021.100277), does not require digestion and contains 18 types of amino acids and several other beneficial nutrients. Therefore, we hypothesized that adding ED to the treatment regimen could help to improve intestinal absorption by maintaining villi in the small intestine,^18^ potentially preventing OM development and improving chemotherapy adherence.

An initial clinical study (EPOC) in patients with esophageal cancer (EC) treated with DCF confirmed the feasibility of administering an ED (160 g/day).^19^ Notably, in that study, we constructed a central review system to judge the oral cavity status, in which oral and maxillofacial surgeons at each institution examined the oral cavity, took photographs, and transmitted them to a central data server. The OM was then graded by a dental specialist experienced in dental oncology, unaffiliated to the participating medical institutions and unaware of each patient’s background.^19^ In an accompanying multicenter, prospective, observational cohort study, the rates and grade of OM determined by this central review system were determined to be significantly higher than those recorded by non-specialist general physicians or medical staff.^20^ Overall, our preliminary data showed that among EC patients treated with DCF, the incidence rates of grade ≥2 OM (using the central review system) were 12.5% in patients receiving an ED and 33.3% in patients not receiving an ED.^20^

We have now conducted a phase III, multicenter, randomized, controlled trial (EPOC 2) to elucidate the preventive effect of an ED against OM in patients with EC receiving DCF therapy using the central review system.

## Patients and methods

### Study design

EPOC 2 was a phase III, multicenter, randomized, controlled, parallel-group study conducted at 16 institutions in Japan from 5 January 2017 to 28 December 2020. The study was registered with the Japan Registry of Clinical Trials (identifier: jRCTs071180029) and the University Hospital Medical Information Network (identifier: UMIN 000025412) and was conducted in accordance with the ethical principles laid out by the Declaration of Helsinki. The independent ethics committee of each participating institution approved the protocol. All patients provided written informed consent before commencing study-related procedures.

### Patients

Eligible patients aged ≥20 years at the time of registration with histologically or cytologically confirmed squamous cell carcinoma, adenosquamous cell carcinoma, or Siewert type I adenocarcinoma of the esophagus were enrolled by the study investigators. Patients agreed to initiate DCF therapy either as preoperative chemotherapy (for stage II/III EC), for unresectable disease (clinical T4 cases or distant metastasis), or as the first treatment after recurrence for recurrent disease. Disease staging was defined according to the International Union Against Cancer tumor-node-metastasis (TNM) classification system, 7th edition.^21^

In addition, patients were required to have an Eastern Cooperative Oncology Group performance status of 0-1; adequate liver, bone marrow, renal, and cardiovascular function [serum bilirubin ≤1.2 mg/dl, a leucocyte count of 4000-12 000/mm^3^, a neutrophil count ≥2000/mm^3^, serum aspartate aminotransferase and alanine aminotransferase levels ≤100 IU/l, a platelet count ≥10 × 10^4^/mm^3^, hemoglobin ≥8.0 g/dl, and creatinine ≤1.2 mg/dl (or creatinine clearance > 50 ml/min)]; and a dysphagia score ≤2. Dysphagia was scored according to a system previously developed for use in patients with EC^22^ as a modification of an older scoring system,^23^ where 0 = able to eat a normal diet, 1 = able to eat some solid food, 2 = able to eat semi-solid food only, 3 = able to swallow liquids only, and 4 = complete dysphagia.

The major exclusion criteria included symptomatic infectious disease; symptomatic peripheral neuropathy; diabetes mellitus controlled by insulin; pregnancy or lactation; hypersensitivity to DCF, the ED, or polysorbate 80-containing formulations; disorders of amino acid metabolism; habitual use of steroids; severe interstitial pneumonia, large quantities of pleural effusion, or ascites; symptomatic bone or brain metastases; presence of OM at registration; and recurrent disease where the total dose of cisplatin in previous treatments exceeded 210 mg/m^2^.

### Treatment

After confirmation of eligibility, patients were randomly assigned (1 : 1 ratio) by the central data center using the dynamic allocation method via a web-response system to receive two cycles of DCF with (group A) or without (group B) the ED. The randomization system was developed by e-Trial Co., Ltd (Tokyo, Japan) and stratified by institution, age <70 or ≥70 years, and preoperative or unresectable/recurrent disease. A minimization method was applied to eliminate bias during randomization. Study personnel did not have access to the randomization data other than to obtain treatment assignments for each participant. Neither the patients nor investigators were blinded to treatment and group B did not receive a placebo in place of the ED.

In group A, patients were administered the ED starting 7 days before chemotherapy initiation and continuing for 56 days from day 1 of the first cycle of DCF. The ED was orally administered on a daily basis (160 g/day) during two cycles of chemotherapy (Supplementary Material S2, available at https://doi.org/10.1016/j.esmoop.2021.100277).

Following treatment allocation, all patients had at least one appointment to discuss oral care and oral hygiene management between 2 weeks and 1 day before starting DCF chemotherapy. Once chemotherapy was begun, only treatment of dental caries was allowed. Current guidelines recommend oral health care and patient education by appropriately trained professionals before starting chemotherapy.^24^ We disseminated a standardized oral hygiene management manual to all participating facilities to ensure that OM differences were due solely to the effects of the ED and to reduce confounding resulting from variations in oral treatment by institution or by clinical specialty.

Patients received two cycles of DCF, in alignment with previous Japanese clinical studies in EC.^25^^,^^26^ Docetaxel (35 mg/m^2^) and cisplatin (40 mg/m^2^) were administered on days 1 and 15, and 5-fluorouracil (400 mg/m^2^) was administered continuously on days 1-5 and 15-19 of each 28-day cycle. All patients were pretreated with the antiemetic granisetron, a neurokinin-1 receptor antagonist, and dexamethasone (8 mg). Dexamethasone was also administered prophylactically for hypersensitivity reactions. The protocol did not allow the use of prophylactic granulocyte colony-stimulating factor. Ciprofloxacin (500 mg × 5 days) was allowed on days 6-10, 20-24, 34-38, and 48-52 to prevent febrile neutropenia.

### Trial endpoints and assessments

The primary endpoint was the incidence of grade ≥2 OM assessed by two specialists from the central review system blinded to allocation group, assessment timing, and background data. Oral and maxillofacial surgeons at each institution photographically imaged the oral cavity on days 1, 15, 29, 43, and 57 of the study (Supplementary Material S2, available at https://doi.org/10.1016/j.esmoop.2021.100277). Six photographs (comprising the posterior surfaces of the upper and lower lips, the right and left buccal mucosa, and the right and left lingual surfaces) were obtained using a digital DSLR camera specialized for intraoral imaging (EOS Kiss X50, Canon, Tokyo, Japan or other camera with similar specifications) and transmitted as a 1-megabyte electronic file to the data server for central review. In addition to the routine imaging, if grade ≥2 OM was diagnosed by the treating physician or if patients reported oral symptoms of grade ≥2, a photographic record was uploaded to the central review system before starting OM treatment. The primary endpoint, however, was calculated based on the central review results, not the individual physician’s judgment or patient self-report.

OM was defined using the Common Terminology Criteria for Adverse Events (CTCAE). Because the CTCAE v4.0 does not describe grading for the objective (visual) scales of OM, we used v3.0 for OM grading (Supplementary Material S3, available at https://doi.org/10.1016/j.esmoop.2021.100277); however, other AEs were evaluated using v4.0.

Secondary endpoints for both groups were the hazard ratio (HR) of occurrence of grade ≥2 OM, changes in body weight, prealbumin, C-reactive protein (CRP), lymphocytes, and AEs (excluding OM). Measurements of weight fluctuation, prealbumin level, and blood biochemistry were conducted on days 1, 8, 15, 22, 29, 36, 43, 50, and 57 of both treatment cycles. In addition, a paper diary was completed by all patients during the study period to self-report ED intake and subjective symptoms.

For group A (receiving the ED), additional endpoints included the occurrence of grade ≥2 OM by ED compliance, the DCF completion rate based on ED compliance, and the investigation of factors correlating with DCF completion.

### Statistical analysis

Based on our prior data,^20^ in this phase III trial, we expected that the clinical incidence rate of OM with ED administration would decrease by 20% compared with the rate in patients without ED. Accordingly, we assumed a null hypothesis with a 33.3% OM occurrence rate for DCF alone and 13.3% for DCF plus ED. Given a two-sided alpha of 0.05 and statistical power of 80%, a minimum of 138 patients were required. Assuming a drop-out rate of 10%, the final sample size was set at 160 patients (80 with ED and 80 without ED).

The efficacy analyses were conducted using the intention-to-treat (ITT) population, defined as all eligible patients who received treatment. Between-group comparisons of OM incidence were carried out using the chi-square test. The OM occurrence rates over time were estimated using the Kaplan–Meier method with the log-rank test; for event comparisons, the HR and two-sided 95% confidence intervals (CIs) were estimated using the Cox proportional hazards regression model. For changes in laboratory test values, between-group comparisons were analyzed by a linear mixed-effects model with patients as a random effect. Fisher’s exact test was used to examine grade ≥2 OM and the DCF completion rate based on ED compliance, the AEs in each group, and the factors correlating with the DCF completion rate. In all cases, *P* values <0.05 were considered to indicate statistical significance. No multiplicity adjustments were carried out. All statistical analyses were carried out using SAS software (v9.4; SAS Institute, Inc., Cary, NC).

## Results

### Patients

At the end of the planned study period, running from 5 January 2017 to 28 December 2020, the number of registered cases had not reached 160. Because the estimated statistical power at that time was 70% or more, however, we opted to discontinue case registration.

**Figure 1:**
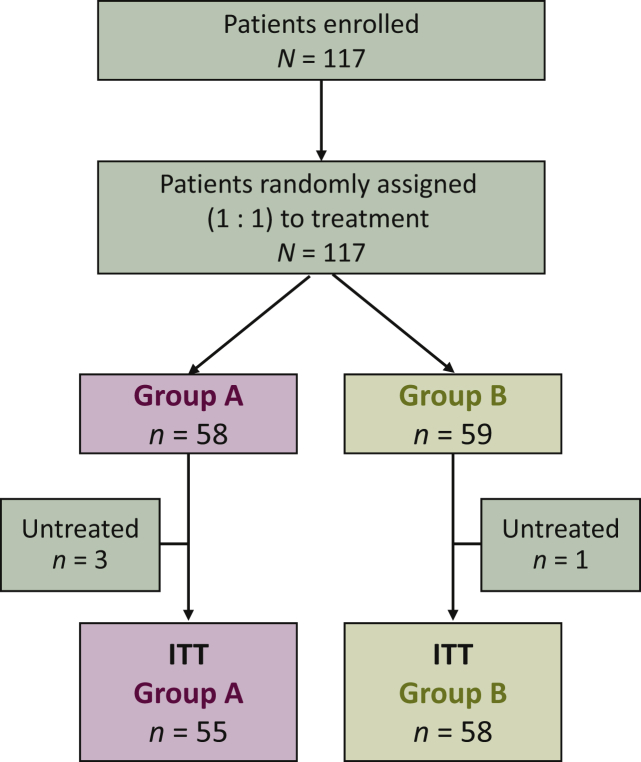
CONSORT diagram. No patients were excluded due to duplicate registration or registration made in error. ITT, intention-to-treat.

Of the 117 patients randomly assigned to treatment, four failed to initiate treatment and were excluded from the primary analysis; therefore, the ITT population included 113 patients (group A, *n* = 55; group B, *n* = 58; Figure 1).

Patient baseline characteristics are summarized in Table 1. There were no significant differences between the two groups. There were also no significant differences between the two groups in the DCF doses administered (docetaxel, *P* = 0.6151; cisplatin, *P* = 0.2929; 5-fluorouracil, *P* = 0.8214).

**Table 1:** Patient characteristics at baseline

Factors	Group A *n* = 55	Group B *n* = 58	*P* value
Sex			0.2639^a^
Male	43 (78)	50 (86)	
Female	12 (22)	8 (14)	
Age, years			
Median (min, max)	68 (44, 86)	68 (34, 83)	0.6731^b^
<70	33 (60)	33 (57)	0.7380^a^
≥70	22 (40)	25 (43)	
Body surface area			0.4476^b^
Median (min, max)	1.6 (1.2, 2.1)	1.6 (1.2, 2.1)	
Cancer treatment			0.8725^a^
Neoadjuvant	34 (62)	35 (60)	
Unresectable/recurrence	21 (38)	23 (40)	
ECOG performance status			0.6678^a^
0	31 (56)	35 (60)	
1	24 (44)	23 (40)	
Location of main lesion			0.4498^a^
Cervical esophagus	2 (4)	4 (7)	
Thoracic esophagus	52 (95)	51 (88)	
Upper	6	6	
Middle	28	21	
Lower	18	24	
Abdominal esophagus	1 (2)	3 (5)	
Pathology			0.9811^a^
Squamous cell carcinoma	48 (87)	50 (86)	
Adenocarcinoma	6 (11)	7 (12)	
Adenosquamous cell carcinoma	1 (2)	1 (2)	
Stage			0.4479^a^
IA	0	1 (2)	
IB	0	1 (2)	
IIA	6 (11)	7 (12)	
IIB	6 (11)	9 (16)	
IIIA	11 (20)	9 (16)	
IIIB	11 (20)	4 (7)	
IIIC	11 (20)	14 (24)	
IV	10 (18)	13 (22)	

Data are shown as *n* (%) unless otherwise specified.

ECOG, Eastern Cooperative Oncology Group.

a Calculated using the Chi-square test.

b Calculated using the *t*-test.

### Primary endpoint

**Figure 2:**
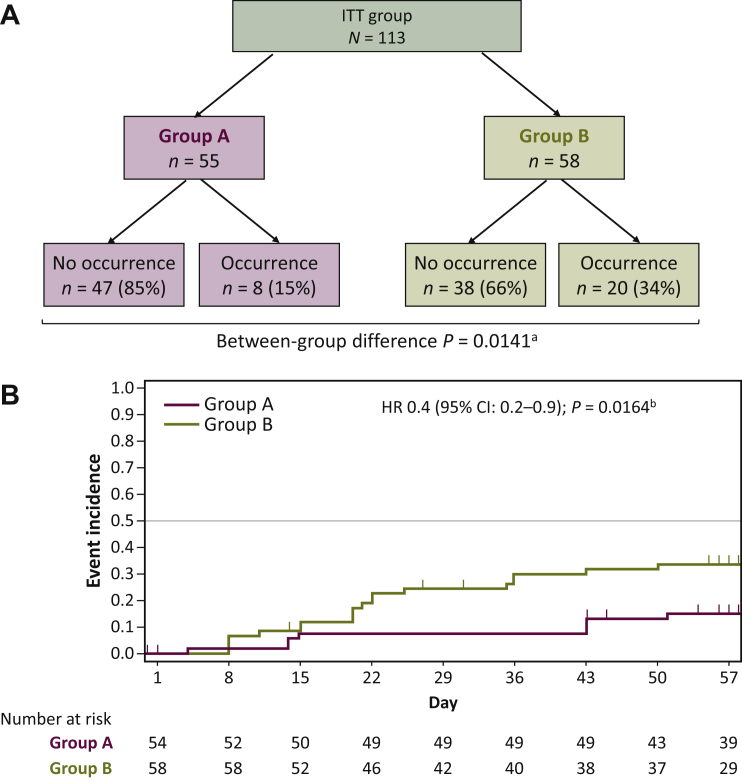
Occurrence of grade ≥2 oral mucositis. (A) Rate of occurrence in each group. (B) Kaplan–Meier diagram of the occurrence over time. CI, confidence interval; HR, hazard ratio; ITT, intention-to-treat. ^a^ Calculated using the Chi-square test. ^b^ Calculated using the log-rank test.

As shown in Figure 2A, the incidence of grade ≥2 OM (by central review) was significantly lower in group A (8/55 patients, 15%) than in group B (20/58, 34%; *P* = 0.0141, chi-square test). In the Kaplan–Meier analysis (Figure 2B), there was a significant difference in the incidence of grade ≥2 OM between the groups, especially around day 22 [HR, 0.4 (95% CI 0.2-0.9); *P* = 0.0164].

Grade 0 OM was recorded in 9/55 (16%) patients in group A and 4/58 (7%) in group B; grade 1 OM was recorded in 38/55 (69%) and 34/58 (59%), respectively. There was no statistically significant difference in the OM rates of grade 0 versus ≥1 (*P* = 0.1149, chi-square test). No events of grade ≥3 OM were recorded.

### Secondary endpoints

Secondary endpoints are summarized in Supplementary Material S4, available at https://doi.org/10.1016/j.esmoop.2021.100277. In group A, body weight was maintained while in group B, it declined (*P* = 0.0022). Increases from day 0 in prealbumin were significantly higher in group A than group B (*P* = 0.0203). During the first cycle, CRP levels were significantly lower in group A than group B (*P* = 0.0338). There were no differences between the two groups regarding changes in lymphocyte count throughout the two DCF cycles (*P* = 0.3472).

Regarding the incidence of AEs overall, 54/55 (98%) patients in group A had at least one AE, and 57/58 (98%) in group B reported at least one AE (*P* = 1.0000) (Table 2). Regarding the incidence of grade ≥3 non-hematologic toxicity, there was no significant difference between groups [group A, 10/55 (18%); group B, 7/58 (12%); *P* = 0.3636]. Elevated alanine aminotransferase was significantly more common in group A than group B (*P* = 0.0311).

**Table 2:** Adverse events (other than oral mucositis) in each group and between-group differences

Adverse events^a^	Group A *n* = 55			Group B *n* = 58			*P* value^b^		
AE, grade	All	≥2	≥3	All	≥2	≥3	All	≥2	≥3
Overall	54 (98)	49	27	57 (98)	50	26	1.000	0.6419	0.6499
Hematologic toxicity									
Overall	50 (93)	37	16	52 (90)	44	25	0.7439	0.3854	0.1391
Anemia	48 (87)	22	4	50 (86)	22	6	0.8674	0.8216	0.7433
Leucopenia	30 (55)	20	3	43 (74)	33	14	0.0295	0.0288	0.0055
Lymphocytes decreased	26 (47)	21	7	26 (45)	19	7	0.7944	0.5468	0.9155
Thrombocytopenia	22 (40)	1	1	23 (40)	4	0	0.9701	0.3646	0.4867
Neutropenia	21 (38)	19	6	41 (71)	36	21	0.0005	0.0034	0.0016
Non-hematologic toxicity									
Overall	54 (98)	36	10	57 (98)	33	7	1.0000	0.3511	0.3636
Hypoalbuminemia	51 (93)	14	0	48 (83)	8	0	0.1079	0.1176	—
Anorexia	39 (71)	6	2	31 (53)	7	0	0.0560	0.8468	0.2347
Alopecia	32 (58)	16	0	35 (60)	18	0	0.8150	0.8219	—
Constipation	29 (53)	2	0	30 (52)	2	0	0.9150	1.0000	—
ALT	27 (49)	3	2	17 (29)	1	1	0.0311	0.3552	0.6117
Malaise	17 (31)	3	0	21 (36)	3	0	0.5513	1.0000	—
AST	17 (31)	2	1	10 (17)	1	0	0.0886	0.6117	0.4867
Serum creatinine	15 (27)	2	1	15 (26)	2	0	0.8652	1.0000	0.4867
Nausea	11 (20)	2	1	11 (19)	3	0	0.8896	1.0000	0.4867
Extremity edema	9 (16)	0	0	9 (16)	1	0	0.9022	1.0000	—
Diarrhea	7 (13)	3	1	7 (12)	2	2	0.9155	0.6736	1.0000
Fever	7 (13)	1	0	5 (9)	1	0	0.4788	1.0000	—
Hyponatremia	6 (11)	5	5	3 (5)	3	3	0.3131	0.4823	0.4823
Fatigue	5 (9)	1	0	7 (12)	0	0	0.6075	0.4867	—
Peripheral sensory neuropathy	3 (5)	0	0	3 (5)	0	0	1.0000	—	—
Vomiting	3 (5)	0	0	2 (3)	0	0	0.6736	—	—
Pneumonitis	2 (4)	2	0	0 (0)	0	0	0.2347	0.2347	—
Febrile neutropenia	1 (2)	1	1	2 (3)	2	2	1.0000	1.0000	1.0000
Esophageal candidiasis	1 (2)	0	0	1 (2)	0	0	1.0000	—	—
Stomach stasis	1 (2)	1	0	0 (0)	0	0	0.4867	0.4867	—
Dysgeusia	0 (0)	0	0	2 (3)	1	0	0.4959	1.0000	—
Buccal mucosa candida	0 (0)	0	0	1 (2)	1	0	1.0000	1.0000	—
Enteritis	0 (0)	0	0	1 (2)	0	0	1.0000	—	—
White matter encephalopathy	0 (0)	0	0	0 (0)	0	0	—	—	—
Esophageal fistula	0 (0)	0	0	0 (0)	0	0	—	—	—

Data in the table are *n* or *n* (%).

AE, adverse event; AST, aspartate aminotransferase; ALT, alanine aminotransferase.

a Sorted by incidence in group A.

b The chi-square test was used for comparisons, except when the minimum expected frequency was <5, in which case Fisher’s exact test was used.

Regarding hematologic toxicity, all-grade leucopenia and neutropenia occurred significantly more frequently in group B than group A (leucopenia, *P* = 0.0295; neutropenia, *P* = 0.0005). Similarly, grade ≥2 and grade ≥3 leucopenia (*P* = 0.0288 and *P* = 0.0055, respectively) and neutropenia (*P* = 0.0034 and *P* = 0.0016, respectively) were significantly more frequent in group B than group A.

For group A (receiving ED), the occurrence of OM grade ≥2 and the DCF completion rate by ED compliance rate are shown in Table 3. Of the 45 patients with 100% ED compliance, 6/45 (13%) had grade ≥2 OM, and of the 10 patients with ED compliance <100%, 2/10 (20%) had grade ≥2 OM (*P* = 0.6273). DCF completion was 100% (45/45) in patients with an ED compliance of 100% and 70% (7/10) in patients failing ED completion (*P* = 0.0046). The DCF completion rate in group B was 93.1% (54/58), which is lower than in group A [94.5% (52/55)]. In groups A plus B the DCF completion rate was also significantly higher in the ED completion group (45/45) than the ED non-completion group (61/68) (i.e. a combination of patients in group A who did not complete ED and group B who were not administered ED); *P* = 0.0406.

**Table 3:** Compliance rates for the elemental diet, and incidence of oral mucositis (grade ≥ 2) and DCF completion (group A)

Elemental diet compliance rate, %	Patients, *n*	OM of grade ≥2, *n* (%)	DCF completion, *n* (%)
100	45	6 (13)	45 (100)
≥90	5	1 (20)	4 (80)
≥80	2	0	1 (50)
<80	3	1 (33)	2 (67)
100	45	6 (13)	45 (100)
< 100	10	2 (20)	7 (70)
*P* value^a^		0.6273	0.0046

DCF, docetaxel, cisplatin, and 5-fluorouracil; OM, oral mucositis.

a Calculated using Fisher’s exact test.

The investigation of factors correlating with DCF completion found that only 100% compliance of ED was significantly correlated (*P* = 0.0046; Supplementary Material S5, available at https://doi.org/10.1016/j.esmoop.2021.100277).

## Discussion

During anticancer therapy, OM is commonly underestimated^1^ because the accuracy of OM evaluation depends on the ability to survey the oral cavity adequately using specialist instrumentation.^27^ In this EPOC 2 study, we examined whether an ED could prevent OM during chemotherapy using a central review system in which blinded dental experts used photographic images of the oral cavity to ascertain OM occurrence. This technique has allowed us to eliminate bias resulting from the judgment of non-specialists and self-reported patient symptomatology. Our results indicated that in the group of patients receiving the ED, the incidence of grade ≥2 OM was significantly suppressed.

Grade ≥2 visual findings of OM were set as the primary endpoint in this study because most patients were reported to have symptoms correlating with a visual appearance at this grade.^28^^,^^29^ Indeed, our study also found a significant correlation between the clinical findings and the symptoms of grade ≥2 OM (*P* = 0.0023). It could be argued that grade ≥2 visual findings (per central review) should not be included without corresponding functional symptoms. The onset of OM causes infections, however, and reduces the tolerability of anticancer drugs.^1^ Thus, reliance on patient self-reported symptoms may not fully capture the impact of OM on the overall clinical situation. Notably, no patients in our study were diagnosed with grade ≥3 OM per central review, likely because interventions were initiated at the time of grade 2 diagnosis, preventing further progression.

In OM, the DNA of cells in the mucous membrane is directly damaged by cytotoxic drugs by a process involving the tumor necrosis factor-α (TNF-α) signaling cascade and the action of interleukin (IL)-6 and IL-1β. The result is mucosal ulceration with increased susceptibility to bacterial infections. Keratinocytes release transforming growth factor-beta 1 to repair the mucous membrane by inhibiting the cell cycle, recruiting leucocytes, and improving the damaged signaling. Re-epithelialization begins with fibroblasts creating new cells from the pseudomembrane.^30^, ^31^, ^32^, ^33^

Palifermin is a recombinant human keratinocyte growth factor 1 that decreased severe OM in patients undergoing post-operative chemoradiotherapy for head and neck cancer.^34^ Treatment costs are high, however, and palifermin may support cancer cell growth, making it unsuitable for OM management.^1^ Other potential OM treatments include cryotherapy, morphine, mouthwashes containing analgesics, and Chinese herbal medicines; however, these aim to reduce symptoms rather than prevent them.^24^^,^^35^ In the real-world setting, OM prevention is key; once symptoms have developed, patients can experience considerable distress and intense pain, reducing their willingness to eat and quality of life, and requiring the use of painkillers and discontinuation of chemotherapy.^36^

Interest in an ED to prevent OM stemmed from the discovery that glutamine could reduce symptom severity and duration in patients receiving cytotoxic drugs.^15^ Glutamine is an essential fuel for rapidly dividing mucosal cells and has been linked to systemic immunity,^37^ and the growth of lymphocytes, fibroblasts, and enterocytes.^38^ Notably, when glutamine consumption exceeds its synthesis (for example, during major surgery or chemotherapy), it becomes a conditionally essential amino acid.^39^ In meta-analyses, however, no significant differences in the risk of developing OM were found between groups of cancer patients who did or did not receive glutamine supplementation.^16^ The glutamine doses used (10-30 g/day) were chosen to match *de novo* synthesis rates in muscle cells. Previous studies had indicated that comparable doses were able to improve outcomes in patients with severe burns.^40^ Because of the rapid cell cycling of the mucosal epithelium, in addition to chemotherapy-induced villus atrophy within the small intestine, absorption may not catch up even after large-scale glutamine supplementation.^17^^,^^18^ Thus, administration of glutamine alone will be insufficient to prevent the development of OM.

The use of an ED in addition to a chemotherapeutic regimen may be more beneficial than glutamine alone because the ED does not require digestion, and its constituents are easily absorbed, even when the intestinal villi are compromised by cytotoxic treatment.^31^^,^^41^ Moreover, in our prospective feasibility study,^18^ even if the amounts of supplemented glutamine were the same, patients receiving an ED demonstrated significant OM suppression and maintenance of small intestinal villi. Thus, it appears that an ED can also maintain intestinal viability, further increasing the ability to absorb nutrients. These data are consistent with a preclinical study in which ED intake was able to maintain intestinal villi viability against 5-fluorouracil.^42^

The ED contains various amino acids, several of which have been reported to affect inflammation and tissue repair, assisting in suppressing OM. Histidine, an essential amino acid in mammals, appears to have anti-inflammatory^43^ and antioxidant effects.^44^^,^^45^ Therefore, histidine may protect against the free radical formation induced by cytotoxic treatment.^46^ Tryptophan has an inhibitory effect on intestinal antigen permeability,^47^ and glycine can prevent overexpression of IL-1β and TNF-α.^48^ Isoleucine may be able to bolster the mucosal barrier defenses,^49^ and leucine has been shown to activate complex 1 of the mammalian target of rapamycin, an important regulator of T cell proliferation and differentiation.^50^ Arginine stimulates cell migration and may enhance intestinal restitution; it also affects cell signaling and cell proliferation via its metabolites and is essential for wound healing.^51^ Serine, threonine, and proline contribute to the formation of intestinal mucin.^52^ These benefits, however, have only been examined in animal models. We estimate that the dosage of ED used in this study (160 g/day) is equivalent to about one-third of the daily amino acid intake of healthy adults (Supplementary Material S6, available at https://doi.org/10.1016/j.esmoop.2021.100277). Although we presume that the effects of ED are related to increases in amino acid concentrations,^18^ further research on the effects of each amino acid on humans, particularly those receiving anticancer therapy, is desired.

In this study, body weight was maintained in the ED group to a significantly greater extent than the non-ED group. Conversely, loss of appetite tended to be higher in the ED group (*P* = 0.0560). A meta-analysis of oral supplementation during chemotherapy did not find strong evidence to support a direct impact on the maintenance of body weight.^53^ Despite this, previous studies of the ED have shown that early administration after gastric cancer surgery was able to reduce weight loss 1 year later,^54^ and that ED administration was associated with significant lean body mass recovery in patients undergoing chemotherapy for EC.^55^ We speculate that weight loss was suppressed by ED intake, especially while patients received invasive therapy like surgery or chemotherapy, as it contributed to the retention of intestinal villi. Thus, the absorption capacity was maintained.

Our data showed that CRP in group A was significantly lower than group B during the first DCF cycle, and this tendency was maintained throughout two cycles. CRP synthesis is known to be rapidly up-regulated by cytokines (e.g. TNF-α and IL-6) originating at the site of inflammation and tissue damage.^56^^,^^57^ The cytotoxic chemotherapy damages not only the oral cavity but also the mucosal lining of the gastrointestinal tract.^31^ A similar induction in pro-inflammatory cytokines has been observed in inflammatory bowel diseases,^58^ and an ED was shown to have a suppressive effect on the levels of mucosal inflammatory cytokines in Crohn’s disease.^59^ There is no evidence that ED is superior to other oral, enteral nutritional supplements for Crohn’s disease and this study did not compare the OM-suppressing effect of ED with that of other nutritional supplements. ED was shown to be more beneficial for remission in Crohn’s disease than corticosteroids, mainly because it improves luminal lesions.^60^ Thus, ED is recommended for Crohn’s disease induction therapy in Japanese guidelines.^61^ ED suppressed the levels of mucosal inflammatory cytokines,^59^ specifically reducing the production of cytokines like TNF-α and IL-6 in the mucosa in patients with Crohn’s disease.^62^ We can postulate that pathological mechanisms underlying the intestinal mucosal damage in Crohn’s disease and anticancer drugs could be similar, underpinning the healing potential of ED in both conditions.

In this study, hematologic toxicities were significantly lower in the ED group. The concept of ‘febrile mucositis’ in relation to chemotherapy has recently been advocated. Patients who receive cytotoxic therapy experience mucosal barrier injury, triggering resident microorganisms to cause bloodstream infections by disrupting the highly regulated host-microbe interactions, resulting in strong inflammatory reactions via cytokine release.^63^ In this paradigm, ‘neutropenia’ and ‘mucositis’, resulting from chemotherapy, should be recognized as being complementary outcomes. This novel concept may support the results of this study, in which ED-treated patients tended to have reduced neutropenia, decreased inflammation of mucosa, and lower CRP levels.

In group A, the proportion of patients with DCF completion was significantly higher in patients with 100% ED compliance than in patients failing ED completion. The DCF completion rate in group B was lower than in group A. Additionally, in all patients, the DCF completion rate was significantly higher in patients with 100% ED completion than in patients who did not complete ED. Of the factors correlating with DCF completion, only 100% compliance of ED was significant. From this, it is clear that improving compliance with the ED is a key factor in improving patients’ clinical outcomes. For effective implementation of the ED in oncology settings, consideration must be paid to the use of flavor, the shape and texture of the product, and the daily timing and duration of intake, balanced against ordinary meals.

One of the study’s potential limitations is a shortfall in the sample size during the scheduled registration period; however, the statistical power calculated for the 113 patients in the ITT population was estimated to be 71.2%, and we consider that this provides a reasonable level of evidential reliability. Furthermore, an interim analysis was not conducted before it was decided that case accrual would be discontinued. Additionally, the blinding of the central reviewers reduces the risk of bias and maintains study integrity, as knowledge of treatment group and participants’ background was restricted during the study period. Smoking and drinking are risk factors for both EC^64^ and oral diseases such as OM,^65^ and many patients with EC are smokers and/or heavy drinkers. This study is meaningful because it investigated patients with EC, who are also likely to have OM, and who were treated with DCF, which increases the risk of developing OM. The lack of an ED placebo for group B and non-standardization of the caloric intake between groups are other limitations. The production of a suitable placebo, however, was technically and ethically problematic. The study design required that we use standard chemotherapy management procedures in group B, including normal dietary intake. Again, we consider that using a central review system outweighs these limitations as the study outcome was not reliant upon patient self-reported symptomology or heterogeneous physician interview techniques. Finally, this study had a single primary endpoint, and secondary endpoints were considered exploratory, so adjusting for multiplicity may not have been necessary. This study was conducted at 16 institutions in Japan and as such may not be generalizable to other study populations.

This study is unique among prospective studies evaluating supportive care outcomes for chemotherapy patients because the primary endpoint was an objective measure rather than subjective criteria (e.g. pain, nausea, loss of appetite, malaise, and numbness) often evaluated in other studies. These phase III study findings show that OM was prevented with the addition of an ED to the management regimen and provide encouraging evidence that may help improve the management of OM among patients with cancer receiving chemotherapy.
