# Supplementary material for: Effects of an elemental diet to reduce adverse events in patients with esophageal cancer receiving docetaxel/cisplatin/5-fluorouracil: a phase III randomized controlled trial—EPOC 2 (JFMC49-1601-C5)
Source: ESMO Open. 2021 Oct 6;6(5):100277. doi: 10.1016/j.esmoop.2021.100277 (PMC8511839; doi:10.1016/j.esmoop.2021.100277)
Supplement: Supplementary Material [file mmc1.docx]

**SUPPLEMENTARY MATERIALS (ONLINE-ONLY)**

Supplementary Data S1. Table showing the composition of Elental^®^ 80g (300 kcal)

Supplementary Data S2. Diagram showing the chemotherapy regimen and prescribed oral imaging schedule.

Supplementary Data S3. Description of OM grading as per the Common Terminology Criteria for Adverse Events (CTCAE) v3.0.

Supplementary Data S4. Graph showing the changes in body weight, prealbumin, and CRP compared between the two groups

Supplementary Data S5. Table showing the factors correlating with DCF completion rate.

Supplementary Data S6. Table showing the approximate amino acid levels in the ED compared with the daily intake of healthy adults.

**Supplementary Data S1. Composition of Elental^®^ 80g (300 kcal)**

| **Component** | **Quantity** |
| --- | --- |
| Dextrin | 63.41 g |
| Lipid (Soybean oil) | 0.51 g |
| Nitrogen (Amino acid) | 13.14 g |
| Amino acids | |
| L-Isoleucine | 642 mg |
| L-Leucine | 899 mg |
| L-Lysine hydrochloride | 888 mg |
| L-Methionine | 648 mg |
| L-Phenylalanine | 871 mg |
| L-Threonine | 523 mg |
| L-Tryptophan | 151 mg |
| L-Valine | 701 mg |
| L-Histidine monohydrochloride monohydrate | 501 mg |
| L-Arginine hydrochloride | 1125 mg |
| L-Alanine | 899 mg |
| L-Aspartate Mg•K | 1036 mg |
| Na•L L-Aspartate monohydrate | 867 mg |
| L-Glutamine | 1932 mg |
| Glycine | 505 mg |
| L-Proline | 630 mg |
| L-Serine | 1159 mg |
| L-Tyrosine | 110 mg |
| Vitamins | |
| Vitamin B-1 | 152 μg |
| Vitamin B-2 | 244 μg |
| Vitamin B-6 | 220 μg |
| Vitamin B-12 | 0.72 μg |
| Pantothenic acid | 1.10 mg |
| Niacin | 2.20 mg |
| Folic acid | 44 μg |
| Biotin | 39 μg |
| Choline | 8.56 mg |
| Vitamin C | 7.80 mg |
| Vitamin A | 223.2 μg |
| Vitamin E | 3.3 mg |
| Vitamin D | 1.3 μg |
| Vitamin K | 9 μg |
| Electrolytes |  |
| Sodium | 260.0 mg |
| Potassium | 217.6 mg |
| Magnesium | 40.0 mg |
| Calcium | 157.6 mg |
| Iron | 1.8 mg |
| Copper | 0.2 mg |
| Manganese | 0.3 mg |
| Zinc | 1.8 mg |
| Chlorine | 516.8 mg |
| Iodine | 15.2 μg |
| Phosphorus | 121.6 mg |

Elental was launched in Japan and China in 1981 and 2014, respectively.

**Supplementary Data S2. Chemotherapy regimen and prescribed oral imaging schedule.**

**Supplementary Data S3. OM grading as per the Common Terminology Criteria for Adverse Events (CTCAE) v3.0.**

Objective scales for mucositis in the CTCAE v3.0 are as follows: grade 1, erythema of the mucosa; grade 2, patchy ulcerations or pseudomembranes; grade 3, confluent ulcerations or pseudomembranes, or bleeding with minor trauma; grade 4, tissue necrosis, significant spontaneous bleeding, or life-threatening consequences; grade 5, death. The CTCAE also captures patient-reported ratings for oral mucositis: grade 1, asymptomatic or mild symptoms; grade 2, moderate pain not interfering with oral intake; grade 3, severe pain interfering with oral intake; grade 4, life-threatening consequences.

**Supplementary Data S4. Changes in body weight, prealbumin, and CRP compared between the two groups.**

**
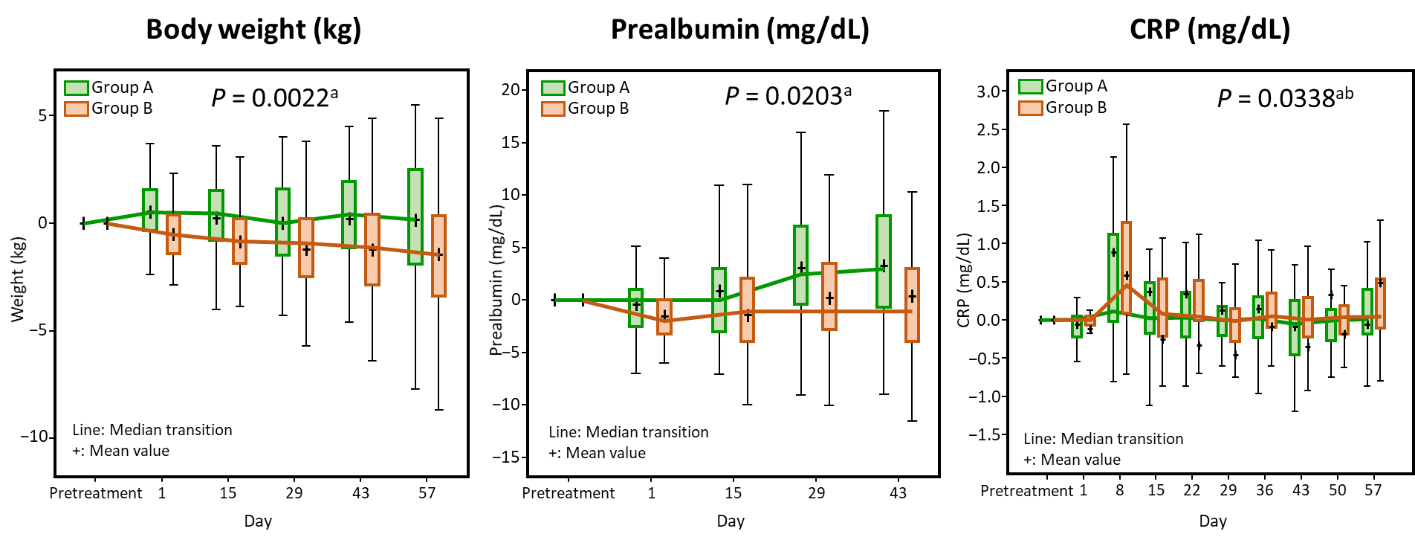
**

^a^ Between-group comparisons were conducted using a linear mixed-effects model with patients as a random effect. CRP data was converted to a logarithmic scale before comparison.

^b^ Calculated only during cycle 1 (days 1–22).

CRP, C-reactive protein

**Supplementary Data S5. Factors correlating with** **DCF completion rate.**

| **Explanatory factors** | | **DCF completed cases, n (%)** | | ***P* value^a^** |
| --- | --- | --- | --- | --- |
|  |  | **100%** | **<100%** |  |
| ED compliance rate | 100% | 45 (100) | 0 (0) | 0.0046 |
|  | < 100% | 7 (70) | 3 (30) |  |
| Sex | Female | 12 (100) | 0 (0) | 1.0000 |
|  | Male | 40 (93) | 3 (7) |  |
| Age, years | < 70 | 32 (97) | 1 (3) | 0.5572 |
|  | > 70 | 20 (91) | 2 (9) |  |
| ECOG PS | 0 | 28 (90) | 3 (9) | 0.2485 |
|  | 1 | 24 (100) | 0 (0) |  |
| Treatment | Preoperative chemotherapy | 32 (94) | 2 (6) | 1.0000 |
|  | Unresectable/recurrence | 20 (95) | 1 (5) |  |
| Pathology | Squamous cell carcinoma | 45 (94) | 3 (6) | 1.0000 |
|  | Other | 7 (100) | 0 (0) |  |
| Stage | Ia-IIIb | 32 (94) | 2 (6) | 1.0000 |
|  | IIIc-IV | 20 (95) | 1 (5) |  |
| Hypoalbumin  (after administration) | Grade 0 | 4 (100) | 0 (0) | 1.0000 |
|  | Grade > 1 | 48 (94) | 3 (6) |  |
| Pre albumin  (Day 0) | < Median | 26 (96) | 1 (4) | 0.5738 |
|  | > Median | 19 (91) | 2 (10) |  |
| Complication  Respiratory disease | Present | 2 (100) | 0 (0) | 1.0000 |
|  | Absent | 50 (94) | 3 (6) |  |
| Complication  Cardiovascular disease | Present | 20 (95) | 1 (5) | 1.0000 |
|  | Absent | 32 (94) | 2 (6) |  |
| All adverse events | Grade > 3 | 25 (96) | 1 (4) | 1.0000 |
|  | Grade < 2 | 27 (93) | 2 (7) |  |
| Hematologic toxicity | Grade > 3 | 13 (100) | 0 (0) | 1.0000 |
|  | Grade < 2 | 39 (95) | 2 (5) |  |
| Non-hematologic toxicity | Grade > 3 | 16 (94) | 1 (6) | 1.0000 |
|  | Grade < 2 | 36 (95) | 2 (5) |  |
| Leukopenia | Grade > 3 | 3 (100) | 0 (0) | 1.0000 |
|  | Grade < 2 | 49 (96) | 2 (4) |  |
| Neutropenia | Grade > 3 | 6 (100) | 0 (0) | 1.0000 |
|  | Grade < 2 | 46 (96) | 2 (4) |  |
| Lymphocyte decreased | Grade > 3 | 7 (100) | 0 (0) | 1.0000 |
|  | Grade < 2 | 45 (96) | 2 (4) |  |
| Thrombocytopenia | Grade > 3 | 1 (100) | 0 (0) | 1.0000 |
|  | Grade < 2 | 51 (96) | 2 (4) |  |
| Fatigue | Grade > 2 | 1 (100) | 0 (0) | 1.0000 |
|  | Grade < 1 | 51 (94) | 3 (6) |  |
| Malaise | Grade > 2 | 3 (100) | 0 (0) | 1.0000 |
|  | Grade < 1 | 49 (94) | 3 (6) |  |
| Anorexia | Grade > 2 | 6 (100) | 0 (0) | 1.0000 |
|  | Grade < 1 | 46 (94) | 3 (6) |  |
| Nausea | Grade > 2 | 2 (100) | 0 (0) | 1.0000 |
|  | Grade < 1 | 50 (94) | 3 (6) |  |
| Vomiting | Grade > 2 | 0 (0) | 0 (0) |  |
|  | Grade < 1 | 52 (95) | 3 (6) |  |
| Diarrhea | Grade > 2 | 3 (100) | 0 (0) | 1.0000 |
|  | Grade < 1 | 49 (94) | 3 (6) |  |

^a^ Calculated using Fisher’s exact test.

DCF, docetaxel, cisplatin, and 5-fluorouracil; ECOG PS, Eastern Cooperative Oncology Group performance status; ED, elemental diet.

**Supplementary Data S6. Approximate amino acid levels in the ED compared with the daily intake of healthy adults.**

| **Amino acid** | **ED (160 g/day), g** | **Healthy adult intake, g/day**^63^ |
| --- | --- | --- |
| Histidine | 1.0 | 2.7 |
| Tryptophan | 0.3 | 1.0 |
| Glycine | 1.0 | 3.6 |
| Isoleucine | 1.3 | 3.5 |
| Leucine | 1.8 | 6.1 |
| Arginine | 2.3 | 4.8 |
| Serine | 2.3 | 3.6 |
| Threonine | 1.0 | 3.2 |
| Proline | 1.3 | 4.5 |

ED, elemental diet.
